# Hog1 Controls Global Reallocation of RNA Pol II upon Osmotic Shock in *Saccharomyces cerevisiae*

**DOI:** 10.1534/g3.112.003251

**Published:** 2012-09-01

**Authors:** Kristen E. Cook, Erin K. O’Shea

**Affiliations:** * Howard Hughes Medical Institute; † Department of Molecular and Cellular Biology, and; ‡ Department of Chemistry and Chemical Biology, Harvard University Faculty of Arts and Sciences Center for Systems Biology, Cambridge, Massachusetts 02138

**Keywords:** MAP kinase, transcription, stress response, osmotic shock, Hog1

## Abstract

When challenged with osmotic shock, *Saccharomyces cerevisiae* induces hundreds of genes, despite a concurrent reduction in overall transcriptional capacity. The stress-responsive MAP kinase Hog1 activates expression of specific genes through interactions with chromatin remodeling enzymes, transcription factors, and RNA polymerase II. However, it is not clear whether Hog1 is involved more globally in modulating the cell’s transcriptional program during stress, in addition to activating specific genes. Here we show that large-scale redistribution of RNA Pol II from housekeeping to stress genes requires Hog1. We demonstrate that decreased RNA Pol II occupancy is the default outcome for highly expressed genes upon stress and that Hog1 is partially required for this effect. We find that Hog1 and RNA Pol II colocalize to open reading frames that bypass global transcriptional repression. These activation targets are specified by promoter binding of two osmotic stress-responsive transcription factors. The combination of reduced global transcription with a gene-specific override mechanism allows cells to rapidly switch their transcriptional program in response to stress.

*Saccharomyces cerevisiae* responds to many environmental perturbations with large-scale changes to its transcriptional program (Gasch *et al.* 2000). For example, when yeast cells are subjected to osmotic shock, the resulting transcriptional response rapidly alters mRNA levels of over one third of the genome (O’Rourke and Herskowitz 2004). During the initial phase of the osmotic shock response, the global transcription rate drops by 50% (Romero-Santacreu *et al.* 2009) due to a general defect in transcription initiation in the first few minutes of stress (Proft and Struhl 2004). Despite this global reduction, transcription of hundreds of genes increases, and the most strongly activated genes are induced over 100-fold within 20 min (Capaldi *et al.* 2008; O’Rourke and Herskowitz 2004), suggesting that cells may have evolved a strategy to prioritize transcription of these genes in the face of harsh conditions.

Transcriptional activation of osmotic stress response genes is coordinated by the MAP kinase Hog1 (Capaldi *et al.* 2008; O’Rourke and Herskowitz 2004). Upon stress, Hog1 is localized to the nucleus (Ferrigno *et al.* 1998) where it regulates the action of several transcription factors (Alepuz *et al.* 2001; Proft *et al.* 2001; Rep *et al.* 2000) and chromatin-modifying enzymes (De Nadal *et al.* 2004; Mas *et al.* 2009). In addition, previous work strongly suggests that Hog1 interacts directly with RNA Pol II in stressed cells. This interaction occurs both *in vitro* (Alepuz *et al.* 2003), as purified Hog1 and RNA Pol II interact in the absence of DNA, and *in vivo* (Alepuz *et al.* 2001; 2003; Proft *et al.* 2006), even in cells lacking Hog1 kinase activity, or when stress-responsive transcription is blocked by deletion of stress responsive transcription factors (Alepuz *et al.* 2003). In stressed cells, Hog1 interacts with components specific to the RNA Pol II initiation complex (Alepuz *et al.* 2003) and is sufficient to recruit RNA Pol II when artificially tethered to a promoter (Alepuz *et al.* 2003). In addition to its role in transcription initiation, Hog1 interacts with the elongating RNA Pol II holoenzyme (Proft *et al.* 2006) and colocalizes with RNA Pol II in the open reading frames (ORF) of some genes (Pokholok *et al.* 2006; Proft *et al.* 2006). A similar behavior has been observed for another stress-induced yeast MAP kinase, Mpk1, which acts as a gene-specific elongation factor during heat shock by blocking attenuation (Kim and Levin 2011). Hog1 regulates chromatin state at specific promoters (De Nadal *et al.* 2004), promotes transcription initiation at specific genes (Alepuz *et al.* 2003), and may, like Mpk1 (Kim and Levin 2011), act as a gene-specific elongation factor, which has been suggested previously (Proft *et al.* 2006). However, it is not clear whether Hog1 plays a role in coordinating the global changes in transcription that accompany osmotic stress (Miller *et al.* 2011; Proft and Struhl 2004; Romero-Santacreu *et al.* 2009), in addition to activating transcription of specific genes.

In this study, we investigate the role of Hog1 in global transcriptional allocation of resources in the early stages of stress. In agreement with previous work (Miller *et al.* 2011; Romero-Santacreu *et al.* 2009), we observe a global reallocation of RNA Pol II upon osmotic shock, in which housekeeping genes lose polymerase occupancy as stress genes are activated. We demonstrate that this reallocation of RNA Pol II depends upon Hog1. We find that depletion of RNA Pol II is the default outcome for highly expressed genes in response to stress and that this depletion is less pronounced in the absence of Hog1. We find that RNA Pol II and Hog1 colocalize to the ORFs of the most highly induced genes during stress. Hog1 colocalizes with RNA Pol II specifically at a set of stress-responsive ORFs that are marked by stress-induced promoter binding of Hog1 and two of its cognate transcription factors, Sko1 and Hot1. We propose a model in which RNA Pol II is preferentially recruited to promoters that contain Sko1 and Hot1 binding sites, allowing recruitment or assembly of a Hog1–RNA Pol II complex and prioritizing transcription of these genes during stress.

## Materials and Methods

### Strains

All strains used in this study are in the W303 strain background (*trp1leu2ura3his3can1 GAL*+ *psi*+), and are listed in supporting information, Table S1. Epitope tags and gene deletions were introduced by transformation with PCR products including auxotrophic or antibiotic markers flanked by the 40 base pairs (bp) of sequence found directly upstream and downstream from the gene, followed by selection on the appropriate medium (Longtine *et al.* 1998; Rothstein 1991). Strains with multiple gene manipulations were constructed by mating the single deletion strains and dissecting the resulting tetrads.

A Quickchange kit (Stratagene) was used introduce amino acid substitutions to generate nonphosphorylatable Sko1 variants. First, 3HA-Sko1 was cloned out of genomic DNA into YCp50, where mutations were introduced. Mutant Sko1 was then reintroduced at the endogenous locus.

Stress-promoted LACZ constructs were derived from pCM173 plasmid (Gari *et al.* 1997), obtained from EUROSCARF. The region between the *Eco*RI site and the 5′ end of LACZ was replaced with a promoter region of interest.

### Chromatin immunoprecipitation

ChIP-seq and ChIP-qPCR experiments were conducted as described previously (Capaldi *et al.* 2008; Johnson *et al.* 2007; Robertson *et al.* 2007), and methods are described in detail in the supporting information. Briefly, cells were grown at 30° in YEPD (or SD for experiments requiring selection for a plasmid), with shaking, to OD_600_ of 0.6. For stress treatment, YEPD supplemented with KCl was added to a final concentration of 0.4 M; for mock-treated cells, the same volume of YEPD was added to cultures. Samples were harvested after 5 min of stress or mock treatment. ChIP against HA-tagged strains was performed with the monoclonal anti-HA antibody 12CA5, and for RNA Pol II ChIP, 1Y26 anti-Rpb3 (Neoclone) antibody was used. For ChIP-seq, ∼10 ng IP material was used to generate each library, following the Illumina protocol for their paired end DNA sample prep kit (v1). For ChIP-qPCR, samples were analyzed on an MX3000p qPCR machine (Stratagene) using primers that amplify a ∼100 bp region surrounding the center of observed binding peaks (primer sequences are listed in Table S2). More detailed methods and analysis of ChIP data are presented in the supporting information. ChIP-seq results have been confirmed by qPCR at selected genes (*PDC1*, *ILV5*, *TDH3*, *ADH1*, *PMA1*, *STL1*, *RTC3*, *HSP12*). Raw data for all ChIP-seq experiments may be obtained from Gene Expression Omnibus (GEO) under accession number GSE38208.

### Inducible LACZ

YCP50 plasmid carrying P_TETO7_-*LACZ*, cloned from pCM173 plasmid (Gari *et al.* 1997), obtained from EUROSCARF, was produced and transformed into a strain expressing rtTA from the *MYO2* promoter, integrated at the *URA3* locus (Kim and O’Shea 2008). Doxycycline concentrations ranging from 0.1 to 2 μg/mL^−1^ were tested to determine an appropriate working concentration that would drive RNA Pol II occupancy of the LACZ construct to a level similar to that of the most highly expressed endogenous genes. Based on approximately 5 hr of growth in rich medium at 30° after addition of doxycycline, 1.25 μg/ mL^−1^ doxycycline was determined to be an appropriate concentration of inducer. For the experiment, cells were grown to saturation overnight, and then diluted to OD_600_ 0.1. At this point, doxycycline was added to induce *LACZ* expression.

### Hog1-occupied ORFs

We determined Hog1 enrichment at each ORF by summing Hog1 ChIP reads that align anywhere in the ORF, subtracting reads obtained in a mock IP, and dividing by reads from sequenced input material. We used three criteria to designate Hog1 ORF occupied genes: Hog1 enrichment; ratio of Hog1 ChIP signal in the presence and absence of stress; and length-normalized Hog1 ChIP signal within the ORF. Cutoff for each criterion was three standard deviations above the genome-wide median. To estimate the false-positive rate, we applied these same criteria to Hog1 ChIP-seq data in the absence of stress (when Hog1 is largely excluded from the nucleus); we identify only one gene that passes all three criteria. We excluded the 5% of genes with the lowest number of input reads to avoid variation in signal due to low alignability. Where two ORFs overlap, we use the nonoverlapping portions to calculate separate enrichment values. Nineteen genes on our list overlap with those identified previously by a lower resolution ChIP-chip assay (Pokholok *et al.* 2006).

### ChIP-seq peak identification

Initial identification of peaks was performed using an implementation of the PeakSeq method (Rozowsky *et al.* 2009) written by Xu Zhou (Zhou and O’Shea 2011). This list of peaks was screened by additional criteria: for one hundred consecutive bases, peaks must be two input standard deviations about the input value at that location and above the overall median input value. This screened list of peaks was then sorted by enrichment over input, and the one hundred highest enrichment peaks were selected for analysis. For this purpose, enrichment was defined as the ratio of IP reads to input reads, with reads summed over the 50 base pairs at the center of each binding peak. Complete lists of all peaks found using PeakSeq are available from Gene Expression Omnibus (GEO) under accession number GSE38208.

### Identification of Sko1 and Hot1 candidate motifs

We conducted a bioinformatics search of the genomic regions enriched in each ChIP-seq experiment using the MEME (Bailey and Elkan 1994) and MAST (Bailey and Gribskov 1998) tools of the web-hosted MEME software suite (Bailey *et al.* 2009). We selected 50 bp regions surrounding the maximum height position of each peak for analysis. During motif discovery, we specified the parameter that each sequence should contain one occurrence of the putative binding motif. We scanned peaks that are bound by Hot1 in stress, bound by Sko1 with higher enrichment pre-stress, and bound by Sko1 with higher enrichment in stress. For discovery of the stress-induced Sko1 binding motif, peaks bound by Sko1 in the absence of stress were used as a set of counterexamples in a discriminatory search. Matches to the discovered Sko1 and Hot1 motifs in the regulatory regions surrounding Hog1-occupied ORFs are listed in File S2 Binding data and motifs found for Sko1 and Hot1 are in general agreement with previous measurements made by ChIP-chip under similar conditions (Capaldi *et al.* 2008).

## Results

### Global reallocation of RNA Pol II during osmotic shock

**Figure 1:**
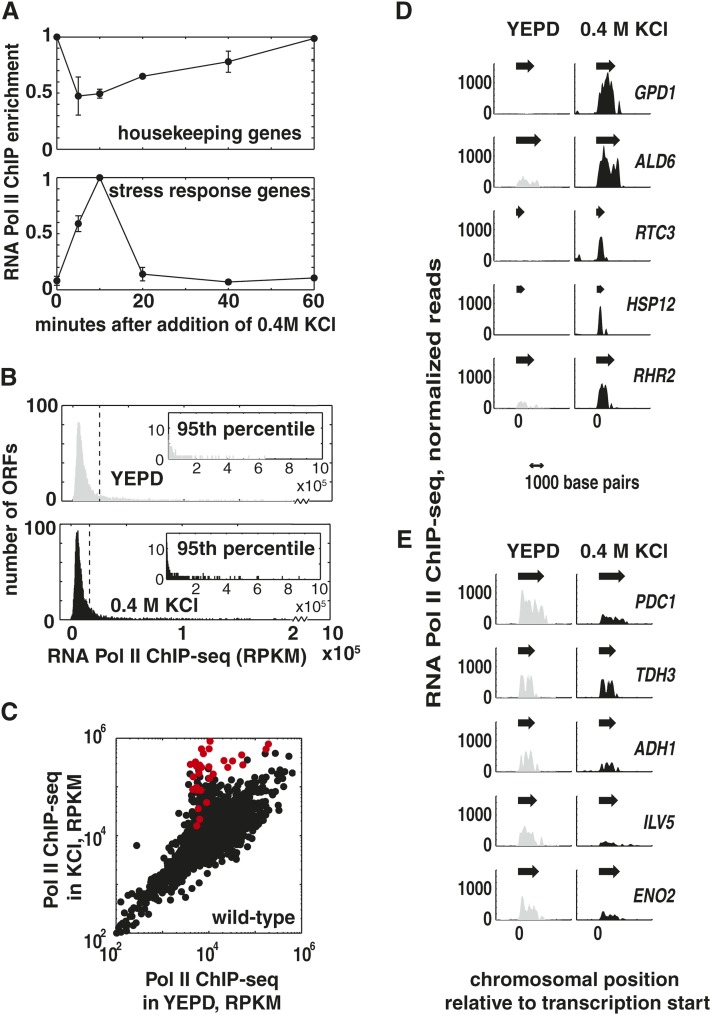
Reallocation of RNA Pol II upon osmotic shock. (A) Dynamics of depletion and recruitment of RNA Pol II in stress were measured by a time course of ChIP-QPCR experiments. Cells in log growth were subjected to osmotic shock by addition of 0.4 M KCl to the growth medium, and samples were collected at the indicated time points. Housekeeping genes with high RNA Pol II occupancy pre-stress are quickly depleted of Pol II upon stress and begin recovering within 10 min (average behavior of *PDC1*, *TDH3*, *ILV5*). Stress-induced genes recruit polymerase upon stress treatment and then rapidly return to low RNA Pol II occupancy (average behavior of *RTC3* and *STL1*). Data shown are the averages of biological replicate experiments. (B) RNA Pol II occupancy was measured by ChIP-sequencing of Rpb3, the third largest subunit of RNA Pol II, in control (mock-treated with YEPD) cells and in cells subjected to 5 min of osmotic shock induced by addition of 0.4 M KCl to the growth medium for 5 min. Histograms show the distribution of RNA Pol II occupancy across all yeast ORFs in each condition. RNA Pol II occupancy is measured at each ORF in reads per kilobase per million (RPKM), after subtracting the input value. Regions of overlap between two or more ORFs were excluded from analysis. Inset plots show the 95^th^ percentile for RNA Pol II occupancy in each condition; the 95^th^ percentile is indicated by a dashed line in the main plot. (C) Scatter plot showing RNA Pol II occupancy during rapid growth in YEPD (x-axis) *vs.* osmotic shock in 0.4 M KCl for 5 min (y-axis). The number of reads that align to each ORF is normalized to RPKM. Each point represents one gene, and points are color-coded to show ORFs that are occupied by Hog1 in response to stress (red points). (D) Distinct sets of genes are top RNA Pol II targets in the presence and absence of stress. RNA Pol II occupancy in rapid growth (gray plots) and after osmotic shock for 5 min in 0.4M KCl (black plots). Values plotted are the number of reads that align at each point along the chromosome, and each ChIP-seq dataset is scaled to one million reads. Arrows indicate the position and direction of each open reading frame. The five highest occupancy ORFs during osmotic shock are shown. (E) The five highest occupancy ORFs during rapid growth in YEPD are plotted, as in (D).

To investigate allocation of transcriptional resources during the early stages of the *S. cerevisiae* osmotic stress response, we measured RNA Pol II location genome-wide by chromatin immunoprecipitation readout by high-throughput sequencing (ChIP-seq) of Rpb3, the third largest subunit of RNA Pol II. We assume that RNA Pol II ChIP signal is proportional to the instantaneous transcription rate, as has been demonstrated previously (Miller *et al.* 2011). Osmotic shock was induced by addition of 0.4 M KCl to the growth medium, and samples were collected after 5 min of stress treatment. We selected this time point to observe the transition from the initial shock phase of the stress response to the induction phase—at 5 min post-stress, the global transcription rate is reduced, but stress responsive genes are actively transcribed (Miller *et al.* 2011; Romero-Santacreu *et al.* 2009; and Figure 1A). Previous work (Miller *et al.* 2011) used dynamic labeling of nascent RNA to characterize a redistribution of RNA Pol II upon osmotic shock that alters transcriptional activity of about 10% of the genome. Using a high-resolution measurement of RNA Pol II occupancy, we observe changes in RNA Pol II occupancy that are consistent with these observations (Figure S1), and additionally, we note that while these stress-induced changes in transcriptional activity affect a relatively small fraction of genes (Figure 1, B and C), this redistribution of RNA Pol II constitutes a major reallocation of the cell’s transcriptional resources (Figure 1C). After 5 min in stress, a set of stress-induced genes shows the highest RNA Pol II occupancy out of all genes in the genome (Figure 1D,), while RNA Pol II occupancy decreases substantially at the genes that had been highly transcribed (Figure 1E).

### RNA Pol II is depleted from a heterologous gene upon osmotic shock

**Figure 2:**
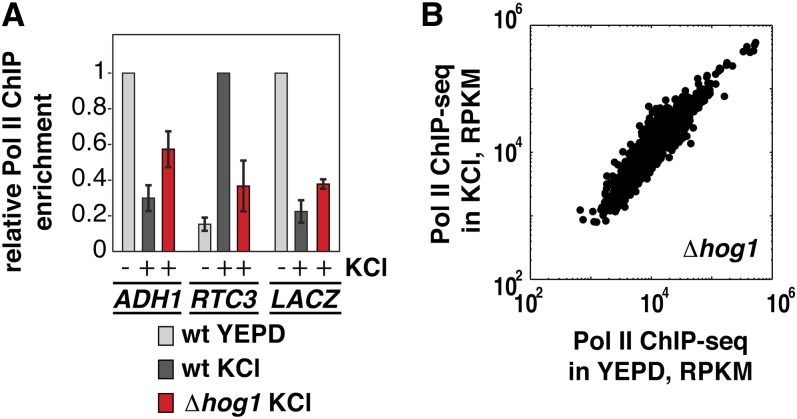
Reallocation of RNA Pol II upon osmotic shock is Hog1-dependent. (A) RNA Pol II is depleted from a heterologous gene upon stress. The TET-ON system was used to drive expression of P_TETO7_-*LACZ*, encoded on a plasmid, to a high level in wild-type cells or in a strain lacking Hog1. Induced cells were then subjected to osmotic shock (as described in (A). RNA Pol II enrichment over a featureless (subtelomeric) region was measured at *LACZ* and at two control genes: *ADH1*, which shows high RNA Pol II occupancy in the absence of stress, and *RTC3*, which shows stress-induced RNA Pol II occupancy. Values plotted are relative RNA Pol II enrichment values for each gene. Error bars show the standard deviation of three biological replicates. (B) Scatter plot showing RNA Pol II occupancy in a *Δhog1* strain during rapid growth in YEPD (x-axis) *vs.* osmotic shock in 0.4 M KCl for 5 min (y-axis). Each point represents one gene. The number of reads that align to each ORF are normalized to reads per kilobases per million (RPKM).

Why are high expression genes depleted of RNA Pol II upon stress, while stress genes are simultaneously induced to a very high level? Perhaps depletion of RNA Pol II is the default response to stress, due in part to the physical effects of stress (Proft and Struhl 2004). To ask if stress-induced depletion of RNA Pol II from highly expressed genes requires specific *cis*-elements, we drove the expression of an exogenous, plasmid-encoded gene (*LACZ*) with an exogenous induction system (Tet-On) and asked if this gene would lose RNA Pol II occupancy in response to stress. We then measured RNA Pol II occupancy in the presence or absence of osmotic shock (5 min in 0.4 M KCl) at *LACZ* and at two control genes (Figure 2A). As expected, RNA Pol II is depleted from the housekeeping gene *ADH1* upon stress, and recruited to the stress-induced gene *RTC3*. Induced *LACZ* behaves like the endogenous housekeeping gene control: RNA Pol II is highly enriched at *LACZ* before stress, and is depleted from *LACZ* upon stress. This result suggests that the default response to stress is depletion of RNA Pol II, and that the coding or regulatory regions of highly transcribed stress-induced genes contain *cis*-elements that enable them to bypass this effect.

### Hog1 controls redistribution of RNA Pol II upon stress

In cells lacking Hog1, the decrease in total transcription that occurs within minutes of stress exposure is initially less pronounced (Romero-Santacreu *et al.* 2009), suggesting a possible role for Hog1 in global transcriptional regulation. To ask if Hog1 is required for depletion of RNA Pol II from highly expressed genes upon stress, we tested the effect of osmotic shock on expression of our heterologous *LACZ* expression system in a strain lacking Hog1 (Figure 2A, red bars). In *Δhog1* cells, a greater proportion of RNA Pol II is retained at the housekeeping gene *ADH1* upon stress treatment, and as expected (Capaldi *et al.* 2008), there is a defect in Pol II recruitment to the Hog1 regulated stress-responsive gene *RTC3*. As in wild-type cells, the heterologous *LACZ* reporter behaves like the endogenous housekeeping gene control, showing less severe depletion of RNA Pol II upon stress in the absence of Hog1 than in wild-type cells. Thus, depletion of RNA Pol II from genes upon stress is partially dependent on Hog1.

To test the influence of Hog1 on the global reallocation of RNA Pol II that we observe in stress (Figure 1C), we measured RNA Pol II occupancy genome-wide in the presence and absence of stress in a *Δhog1* strain. In the absence of Hog1, we no longer observe redistribution of RNA Pol II upon stress (Figure 2B and Figure S2). Thus, the decrease in the overall transcription rate affects all genes similarly in *Δhog1* cells, while wild-type cells actively prioritize transcription of stress-responsive genes over housekeeping genes.

Under osmotic shock conditions, Hog1 is found in a complex with RNA Pol II (Alepuz *et al.* 2003; Proft *et al.* 2006) and is present in some ORFs during elongation (Pokholok *et al.* 2006; Proft *et al.* 2006). To investigate the relationship between high RNA Pol II occupancy and the presence of Hog1 in ORFs during stress, we determined Hog1 localization genome-wide by ChIP-seq in the presence and absence of osmotic stress. We identified 28 ORFs that are enriched for Hog1 during osmotic shock (listed in File S1). These findings are in general agreement with previous work (Pascual-Ahuir *et al.* 2006; Pokholok *et al.* 2006; Figure S3), but by using a higher resolution technique, we are able to more clearly differentiate between Hog1 present in regulatory regions *vs.* coding regions. We find that Hog1 occupies the ORFs that show the highest increase in RNA Pol II occupancy upon stress (Figure 1C, red points), while Hog1 is not present in ORFs that show a decrease in RNA Pol II occupancy in response to stress or in the ORFs of HOG pathway-activated genes that show only modest induction upon stress. Hog1-occupied ORFs show higher RNA Pol II occupancy (median value of 17.1-fold enrichment over input) than HOG pathway-activated genes that are not occupied by Hog1 (median value of 2.4-fold enrichment over input).

### Promoter regions are sufficient to direct Hog1 to ORFs

**Figure 3:**
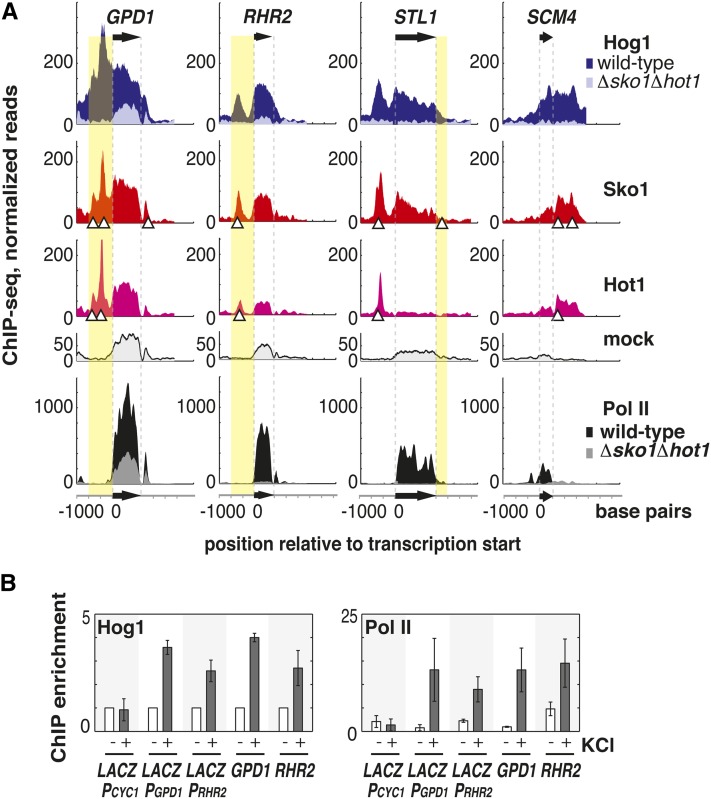
The Hog1 cognate TFs Sko1 and Hot1 target stress-induced genes for transcription by a Hog1–RNA Pol II complex. (A) Colocalization of Hog1, Sko1, and Hot1 at example genes occupied by Hog1 during stress. Lighter colored overlay for Pol II and Hog1 ChIP-seq shows ChIP signal in the *Δsko1Δhot1* strain. Black arrows and dashed gray guidelines mark the position of the *GPD1*, *RHR2*, *STL1*, and *SCM4* ORFs, and white triangles show predicted transcription factor binding sites. Values plotted are the number of reads that align at each point along the chromosome, and each ChIP-seq dataset is scaled to one million reads. Highlighted regions are those shown to be sufficient for Hog1 ORF occupancy. (B) RNA Pol II and Hog1 presence in the *LACZ* ORF driven by three different promoters (defined as 1000 base pairs upstream of each gene): the stress gene promoters P*_GPD1_* and P*_RHR2_*, and P*_CYC1_*, an inactive promoter that serves as a negative control. These promoter-*LACZ* constructs are carried on a plasmid. Hog1 and RNA Pol II ChIP enrichment was measured by qPCR during rapid growth in SD medium lacking tryptophan to maintain the plasmid (bars labeled −) and in response to osmotic shock induced by addition of 0.4 M KCl to the medium (bars labeled +). Enrichment values are normalized by ChIP to *POL1*. For Hog1 enrichment, no-stress values were normalized to one, and stress values show the relative change in enrichment. Error bars show the standard deviation of three biological replicates.

Previous work demonstrates that for at least one Hog1-occupied ORF, the region downstream of the ORF is sufficient to induce Hog1 ORF occupancy (Proft *et al.* 2006). However, it is not clear which *cis*-elements confer this ability, or whether specific transcription factors mediate Hog1 recognition of these *cis*-elements. Hog1 localizes to promoter regions of some target genes (Alepuz *et al.* 2001, 2003; Capaldi *et al.* 2008; Proft and Struhl 2002) as well as ORFs, but the relationship between these two events is unclear, and Hog1 recruitment to ORFs is not thought to be associated with any particular transcription factor (Proft *et al.* 2006). We find that Hog1 presence in ORFs correlates strongly with Hog1 promoter binding. Hog1 shows enrichment (defined as mock-subtracted Hog1 ChIP signal divided by input for the region 1000 bp upstream of each ORF) greater than three standard deviations above the genome-wide median in the promoters of 26 of the 28 occupied ORFs (example genes shown in Figure 3A), compared with 5 of the 231 promoters of HOG pathway-regulated ORFs that are not occupied by Hog1. We also observe Hog1 ChIP enrichment greater than three standard deviations above the genome-wide median in the 3′ regions (50–500 bp downstream) of 18 of the 28 ORFs that are occupied by Hog1, compared with 4 out of 231 of the HOG-pathway regulated ORFs that are not occupied by Hog1.

We were interested in whether promoter regions contain information sufficient to direct Hog1 to ORFs. To address this question, we placed a stress gene promoter upstream of *LACZ* on a plasmid and tested for the presence of Hog1 in the *LACZ* ORF. We find that the promoters of the stress genes *GPD1* and *RHR2* are sufficient to recruit both RNA Pol II and Hog1 to the *LACZ* ORF in a stress-specific manner (Figure 3B). This result, combined with the observation that the region downstream of *STL1* is necessary and sufficient to recruit Hog1 to the *STL1* ORF (Proft *et al.* 2006), suggests that *cis* -regulatory elements contained within these 3′ and 5′ regions are capable of targeting genes for transcription by a complex that includes Hog1.

### ORFs occupied by Hog1 are marked by the presence of Hog1, Sko1, and Hot1 in their regulatory regions

Although Hog1 lacks a DNA binding domain, it is able to associate with promoters through physical interactions with its cognate transcription factors Sko1 (Proft and Struhl 2002) and Hot1 (Alepuz *et al.* 2001, 2003). Of the 28 ORFs occupied by Hog1, 21 require Sko1 and/or Hot1 for activation in response to osmotic shock (Capaldi *et al.* 2008). We hypothesized that these factors may be able to recruit Hog1, and thus the presence of these transcription factors at promoters would correlate with Hog1 recruitment to promoters and ORFs. To obtain a high-resolution map of Sko1 and Hot1 binding within the regulatory regions surrounding Hog1 occupied ORFs, we measured Sko1 and Hot1 binding by ChIP-seq in the presence and absence of stress. At promoters where Hog1 is present, Sko1 and Hot1 colocalize with Hog1 (Figure 3A). Sko1 is present in the promoters of 27 of the 28 Hog1-occupied ORFs, and Hot1 binds within 50 bp of Sko1 at 14 of these promoters. At each of these promoters, the observed transcription factor binding peaks overlap with Hog1 promoter peaks.

**Figure 4:**
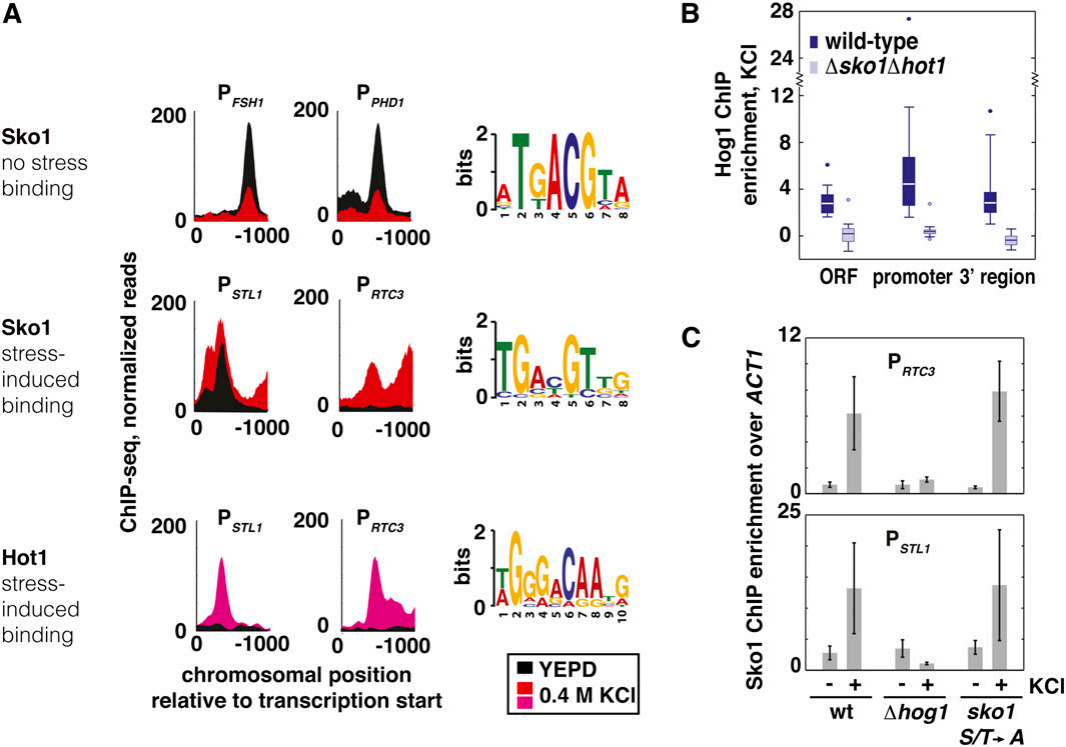
Analysis of binding determinants of Sko1 and Hot1. (A) Binding behavior (left panel) and discovered motifs (right panel) for Hot1 and two classes of Sko1 binding sites observed by ChIP-seq. Sko1 and Hot1 binding is shown in control conditions and in osmotic shock (treatment with 0.4 M KCl for 5 min. Sko1 no-stress peaks show decreased enrichment upon osmotic shock (example peaks from *FSH1* and *PHD1* promoters, top left panel.) Stress-induced Sko1 (center left panel) and Hot1 (bottom panel) binding peaks increase in ChIP enrichment upon stress (example peaks in *STL1* and *RTC3* promoters). A bioinformatics search of 43 pre-stress Sko1 binding sequences reproduces a previously reported Sko1 binding motif, 5′-TGACGT-3′ (Harbison *et al.* 2004). A bioinformatics search of 17 stress-induced Sko1 binding peak sequences reveals a low-information variant of the Sko1 consensus site. A search of 11 high-confidence Hot1 binding peaks allows *de novo* discovery of a candidate motif for Hot1. (B) Hog1 enrichment during stress at ORFs, promoters, and 3′ regions decreases in the absence of Sko1 and Hot1. Box and whisker plot shows Hog1 enrichment in the ORFs, promoters (1000 bp upstream) and 3′ regions (1000 bp downstream) for the set of 28 ORFs occupied by Hog1 during stress in wild-type cells. Enrichment is defined as Hog1 ChIP-seq reads minus mock IP, divided by the input signal. Enrichment values in wild-type (wt) cells are shown in dark blue; and enrichment values for *Δsko1Δhot1* cells are shown in light blue. The edges of each box show the 25^th^ and 75^th^ percentiles, and the line within each box indicates the median. Whiskers extend to the highest and lowest values that are not considered outliers (three standard deviations above or below the median). Outliers are plotted as individual points. (C) Stress-induced Sko1 binding requires Hog1, but not Hog1 phosphorylation, of Sko1. Sko1 binding was measured by ChIP against the HA epitope in wild-type, Δ*hog1*, and *sko1- S108A*, *T113A*, *S126A* (which cannot be phosphorylated by Hog1) strains, all tagged with three copies of the HA tag at the N′ of the endogenous copy of Sko1. ChIP experiments were performed on cells in log-phase growth in rich medium, in the presence or absence of osmotic shock (induced by 5 min in 0.4 M KCl). The data points on the bar graph are the averages of at least three biological replicates, and error bars represent the standard deviation of the measurements.

Using a bioinformatics approach, we identified sequence elements that are overrepresented at sites of Hot1 and Sko1 recruitment in response to stress (Figure 4A). We find that Hog1 colocalizes with Sko1 and Hot1 at overrepresented sites in the promoters, and in some cases, downstream regions of the ORFs that it occupies (Figure 3A, white triangles). These predicted Sko1 and Hot1 binding sites are present in regulatory regions that are sufficient to induce Hog1 ORF occupancy (Figure 3A, highlighted regions). Hog1 recruitment to these regulatory regions depends upon the presence of Sko1 and Hot1; Hog1 is not recruited to promoters, ORFs, or downstream regions in the absence of these two factors (Figures 3A and 4B).

### Hog1 is required for stress-induced Sko1 and Hot1 binding

Although Hog1 phosphorylates Hot1 upon stress, this phosphorylation is not required for Hot1 activity (Alepuz *et al.* 2003). Instead, a physical interaction between Hot1 and Hog1 is required for promoter recognition, and it is Hog1 that recruits the polymerase (Alepuz *et al.* 2003). Sko1 binds constitutively to some genomic locations, while Sko1 binding at other sites is induced by stress (Capaldi *et al.* 2008; Ni *et al.* 2009). While Hog1 is not required for Sko1 binding in the absence of stress, we hypothesize that Hog1 may play a similar role in directing stress-induced binding of Hot1 and Sko1, given that Hog1 colocalizes with Sko1 at stress-induced binding sites (Figure 3A), and Sko1 is not recruited to these sites upon stress in a *Δhog1* strain (Figure 4C). Hog1 phosphorylates Sko1 in response to stress (Proft and Struhl 2002), but we find that this phosphorylation is not required for stress-induced Sko1 binding (Figure 4C), suggesting that Hog1 regulates Sko1 binding as well as Hot1 binding through a sustained physical interaction rather than via phosphorylation. Though Hog1 lacks a DNA binding domain, it partners with Sko1 and Hot1 to recognize specific *cis*-regulatory elements at the ORFs that it occupies during stress. Hog1 interacts directly with RNA Pol II during stress (Pascual-Ahuir *et al.* 2006), and is capable of inducing transcription when tethered to a promoter (Alepuz *et al.* 2003). Based on these observations, we suspect that Hog1 recruits RNA Pol II to the set of genes with nearby Sko1 and Hot1 binding sites and that Hog1 is loaded onto these ORFs along with RNA Pol II during transcription initiation.

**Figure 5:**
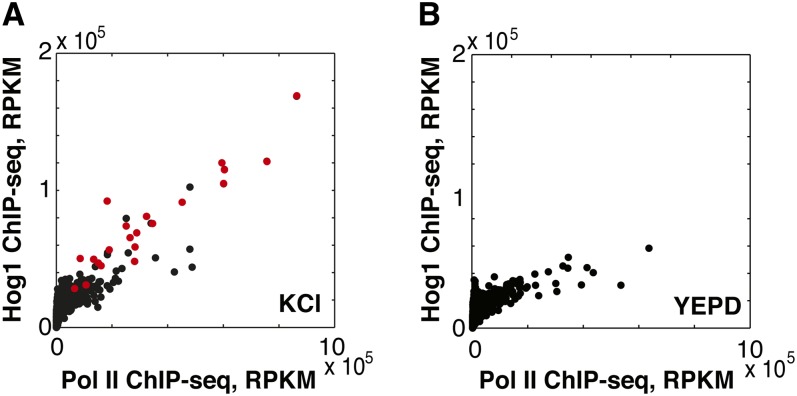
A stress-specific binding mode of Sko1 targets Hog1 to ORFs. (A) In stress, genes bound by a Sko1/Hot1/Hog1 complex (see Table S3) show highest levels of RNA Pol II and Hog1 in ORFs. Scatter plot shows ChIP-seq of Hog1-HA compared with ChIP-seq of Rpb3 after 5 min of osmotic shock with 0.4M KCl. The number of reads that align to each ORF are normalized to reads per kilobases per million (RPKM). Genes that exhibit stress-induced colocalization of Hog1 with Sko1 or with both Sko1 and Hot1 in their regulatory region are shown in red; all other genes are shown in black. (B) Scatter plot shows ChIP-seq of Hog1-HA compared with ChIP-seq of Rpb3 during rapid growth in rich medium.

At the set of genes that show stress-induced recruitment of Sko1, Hot1, and Hog1 to their promoters and/or downstream regions, Hog1 enrichment in ORFs is largely proportional to RNA Pol II occupancy (Figure 5A), consistent with previous reports that Hog1 is part of the elongating RNA Pol II holoenzyme (Proft *et al.* 2006). This relationship between gene expression and Hog1 presence during elongation is specific to stress and does not occur in control conditions (Figure 5B). Taken together, these observations suggest that genes marked by the presence of Hog1, Sko1, and Hot1 in their regulatory regions are selectively transcribed by a Hog1–RNA Pol II complex during stress.

## Discussion

Hog1 and RNA Pol II were known to colocalize at some stress-responsive genes during stress (Pokholok *et al.* 2006; Proft *et al.* 2006), and Hog1 has been shown to associate with components of the elongating RNA Pol II holoenzyme under stress conditions (Proft *et al.* 2006), but the role of this Hog1–RNA Pol II complex in the stress response was not clear. Our results suggest that this complex marks and specifies the most highly induced genes during stress. In response to stress, cells experience a rapid drop in their overall transcription rate. This stress-induced global reduction of transcription may have two causes: (1) a general effect of the physical changes that accompany stress (Proft and Struhl 2004); and (2) a Hog1-dependent signal that increases this effect. Given that Hog1 interacts with the polymerase upon stress (Alepuz *et al.* 2003; Pokholok *et al.* 2006; Proft *et al.* 2006), we suggest that the entry of Hog1 into the nucleus upon stress may serve as this signal. In this model, RNA Pol II either in complex with Hog1 or modified by Hog1 via phosphorylation has reduced affinity for non-stress-responsive genes, allowing Hog1 to briefly hijack some fraction of RNA Pol II to carry out a Hog1-specified transcription program. If high levels of nuclear Hog1 are capable of globally altering transcription, we would expect spurious activation of Hog1 to be detrimental. In fact, overexpression of Hog1, which results in increased levels of nuclear Hog1, is lethal (Wurgler-Murphy *et al.* 1997), and genetic perturbations of upstream signaling that result in constitutive activation of the Hog1 pathway are not viable (Maeda *et al.* 1994, 1995).

The entry of Hog1 into the nucleus upon stress allows two Hog1 cognate transcription factors, Sko1 and Hot1, to bind with Hog1 at *cis*-elements contained in the regulatory regions of stress responsive genes, targeting the Hog1–RNA Pol II complex to these genes. The RNA Pol II-Hog1 complex transcribes highly expressed, stress-induced genes, while avoiding housekeeping genes that were previously highly expressed. The selective targeting of this complex suggests a mechanism by which a set of genes could be exempted from the transient global transcriptional repression that accompanies stress.

The function of polymerase eviction from highly expressed metabolic genes, *e.g.*, *PDC1*, in the early stages of the osmotic stress response may be to reallocate transcriptional resources rather than to modify the expression level of the RNA Pol II–depleted genes. Depletion of RNA Pol II from highly expressed ORFs often does not lead to reduced mRNA levels, as many of the transcripts produced by Pol II depleted genes are in fact stabilized during the stress response (Miller *et al.* 2011; Romero-Santacreu *et al.* 2009). Global depletion of RNA Pol II, coupled with a gene-specific override mechanism, may constitute a strategy that allows the cell to rapidly switch its transcriptional program.

## Supplementary Material

Supporting Information
