# Supplementary material for: Hog1 Controls Global Reallocation of RNA Pol II upon Osmotic Shock in Saccharomyces cerevisiae
Source: G3 (Bethesda). 2012 Sep 1;2(9):1129–36. doi: 10.1534/g3.112.003251 (PMC3429927; doi:10.1534/g3.112.003251)
Supplement: Supporting Information [file supp_2_9_1129__index.html]

Supporting Information 

# Hog1 Controls Global Reallocation of RNA Pol II upon Osmotic Shock in *Saccharomyces cerevisiae*

## Supporting Information for Cook and O'Shea, 2012

**Files in this Data Supplement:**

- Supporting Information - Files S1-S4, Figures S1-S3, and Tables S1-S3 (PDF, 1 MB)
- File S1 - Supporting Results and Discussion (PDF, 66 KB)
- File S2 - Supporting Materials and Methods (PDF, 76 KB)
- Figure S1 - Comparison RNA Pol II occupancy data from this study to data from Miller et al., 2011 (PDF, 101 KB)
- Figure S2 - Comparison of RNA Pol II occupancy across ChIP-seq datasets (PDF, 101 KB)
- Figure S3 - Comparison of Hog1 ORF occupancy data to previous work (PDF, 110 KB)
- Table S1 - List of strains used in this study (PDF, 64 KB)
- Table S2 - List of primers used for ChIP-qPCR (PDF, 65 KB)
- Table S3 - List of genes with Sko1, Hot1 and Hog1 present in regulatory regions (PDF, 49 KB)
- File S3 - RNA Pol II and Hog1 occupancy data from ChIP-seq (.xls, 1 MB)
- File S4 - Motifs for stress-induced binding of Sko1 and Hot1 (.xls, 35 KB)
