# Supplementary material for: Hog1 Controls Global Reallocation of RNA Pol II upon Osmotic Shock in Saccharomyces cerevisiae
Source: G3 (Bethesda). 2012 Sep 1;2(9):1129–36. doi: 10.1534/g3.112.003251 (PMC3429927; doi:10.1534/g3.112.003251)
Supplement: Supporting Information [file supp_2.9.1129_003251SI.pdf]

Supporting Information

Hog1 controls global reallocation of RNA Pol II upon osmotic shock in *Saccharomyces cerevisiae*

[*,§]Kristen E. Cook and [*,§,**,§§]Erin K. O'Shea

[*]Howard Hughes Medical Institute, Harvard University Faculty of Arts and Sciences Center for Systems Biology, Northwest Labs, 52 Oxford Street, Cambridge, MA 02138, USA

[§]Department of Molecular and Cellular Biology, Harvard University Faculty of Arts and Sciences Center for Systems Biology, Northwest Labs, 52 Oxford Street, Cambridge, MA 02138, USA

[**]Department of Chemistry and Chemical Biology, Harvard University Faculty of Arts and Sciences Center for Systems Biology, Northwest Labs, 52 Oxford Street, Cambridge, MA 02138, USA

[§§]To whom correspondence should be addressed. E-mail: Erin_Oshea@harvard.edu.

File S1. Supporting Results and Discussion……………………………………………………………………………………page 2 SI
File S2. Supporting Materials and Methods…………………………………………………………………………………………3 SI
Figure S1 . Comparison RNA Pol II occupancy data from this study to data from Miller et al., 2011. …………………5 SI
Figure S2 .Comparison of RNA Pol II occupancy across ChIP-seq datasets…………………………………………………6 SI
Figure S3. Comparison of Hog1 ORF occupancy data to previous work. ………………………………………………………7 SI
Table S1. List of strains used in this study…………………………………………………………………………………………8 SI
Table S2. List of primers used for ChIP-qPCR…………………………………………………………………………………………9 SI
Table S3. List of genes with Sko1, Hot1 and Hog1 present in regulatory regions………………………………………10 SI
Supporting File Legends………11 SI
File S3. RNA Pol II and Hog1 occupancy data from ChIP-seq.
File S4. Motifs for stress-induced binding of Sko1 and Hot1.
Literature Cited……12 SI

File S1

Supporting Results and Discussion

Stress-induced Sko1 binding predicts Hog1 ORF occupancy

During osmotic shock, the Hog1-regulated transcription factor Sko1 is found in the promoters of 27 of the 28 Hog1 occupied ORFs. Sko1 recognizes a well-defined consensus motif *in vivo* in the absence of stress (Capaldi *et al.* 2008; Harbison *et al.* 2004), and binds to this same motif *in vitro* (Fordyce *et al.* 2010; Nehlin *et al.* 1992). However, the Sko1 consensus motif is absent (evaluated at $P<0.05$) from 20 of the 27 Sko1-bound promoters of Hog1-occupied ORFs. Sko1 was once thought to be constitutively bound to its target promoters, and depending on cellular conditions, recruit either repressive or activating chromatin remodeling factors (Proft and Struhl 2002). However, a genome-wide study of Sko1 binding behavior demonstrates that Sko1 target promoters do not display uniform binding behavior. Instead, genes with Sko1 bound in the promoter fall into two basic classes (Capaldi *et al.* 2008). At one set of genes, Sko1 is bound in normal growth conditions and partially released from the promoter in response to osmotic stress, while for another set of genes, Sko1 binding enrichment increases in response to stress. By using ChIP-seq to generate a high-resolution map of Sko1 binding, we are able to identify individual binding sites that fall into these two classes. We find that stress-induced Sko1 binding correlates with the presence of Hog1 in ORFs, while pre-stress Sko1 binding does not. The presence of a stress-induced Sko1 binding peak in a promoter is predictive of Hog1 presence in promoters and ORFs; genes with stress-induced Sko1 promoter binding peaks show a median Hog1 enrichment (defined by mock-subtracted Hog1 ChIP-seq signal, divided by input) of 5.0 in promoters and 2.8 in ORFs during osmotic shock. In contrast, pre-stress binding of Sko1 in promoters is not predictive of Hog1 presence in promoters (median enrichment of 1.26) or ORFs (median enrichment 0.6) during stress.

File S2

Supporting Materials and Methods

Chromatin Immunoprecipitation

ChIP-seq and ChIP-qPCR experiments were conducted as described previously (CAPALDI *et al.* 2008; JOHNSON *et al.* 2007; ROBERTSON *et al.* 2007). Cells were diluted from an overnight culture to an OD_{600} of 0.1. Cells were grown at 30° C in YEPD, with shaking, to OD_{600} of 0.6. Cultures were split for stress treatment and mock treatment. For stress treatment, YEPD supplemented with KCl was added to ~120 OD units of culture, bringing the final concentration of KCl to 0.4 M; for mock treated cells, the same volume of YEPD was added to cultures. After five minutes in stress, samples were crosslinked with 1% formaldehyde at room temperature for 15 minutes. Crosslinking was quenched with 125 mM glycine for five minutes, and then samples were harvested by centrifugation, washed twice in cold PBS (137 mM NaCl, 2.7 mM KCl, 10 mM sodium phosphate dibasic, 2 mM potassium phosphate monobasic, pH 7.4) and then snap-frozen in liquid nitrogen. Samples were resuspended in 1 mL lysis buffer (50 mM HEPES pH 7.5, 140 mM NaCl, 1 mM EDTA, 1% Triton-X 100, 0.1% Na Deoxycholate) in the presence of protease inhibitors (Roche; Complete) and mechanically lysed by bead beading. Lysates were sonicated (9 cycles of 15 seconds each, power 2 on Misonix 3000) to solubilize chromatin and then clarified by centrifugation. Ten percent of the clarified lysate was reserved to serve as an input control. Clarified lysates (~10 mg for ChIP-seq, ~ 2.5 mg for ChIP qPCR) were incubated with 12CA5 anti-HA antibody, for 2 hours at 4° C before addition of Protein G Dynabeads (Invitrogen). After incubation for 4 hours up to overnight, beads were washed twice with lysis buffer, once with high salt buffer (50 mM HEPES pH 7.5, 500 mM NaCl, 1 mM EDTA, 1% Triton-X 100, 0.1% Na Deoxycholate), once with lysis buffer and once with TE (10 mM Tris, 1 mM EDTA, pH 8). All washes were performed at room temperature for five minutes each, on an end-over-end mixer. Samples were eluted from beads in TE plus 0.67% SDS at 65° C for 30 minutes. Supernatants were removed from beads and incubated overnight at 65° C to break crosslinks. DNA was isolated by digestion of RNA with RNAse A for 2 hours at 37° C, protein digestion with Proteinase K for 2 hours at 55° C, and purified by phenol chloroform extraction and precipitation with ethanol and NaCl. DNA pellets were stored in TE.

For ChIP-seq, ~ 10 ng IP material was used to generate each library, following the Illumina protocol for their paired end DNA sample prep kit (v1). After addition of adaptors, DNA in a size range of 175-300 base pairs was isolated by gel electrophoresis for amplification. Size ranges of prepared libraries were measured on an Agilent Biolanalyzer (average size 225 base pairs) before sequencing on an Illumina Genome Analyzer II (performed by Christian Daly at the FAS Center for Systems Biology Core Facility). Thirty-six base reads were obtained and aligned to the *Saccharomyces cerevisiae* genome using ELAND (Jianwen Zhang performed the alignments). For Hog1 and transcription factor ChIP-seq samples, five million to ten million

reads were obtained. Uniquely alignable sequence tags were mapped to the genome and extended by the average length of the library (minus the adaptor length) in MATLAB. For ChIP-qPCR, samples were analyzed on an MX3000p qPCR machine (Stratagene) using primers that amplify a ~ 100 base pair region surrounding the center of observed binding peaks, or an ~300 base pair region in the coding sequence of the ORF. The sequences of these qPCR primers are listed in Supplemental Table S2.

Identification of stress-induced and pre-stress bound Sko1 binding peaks

Sko1 binding peaks were classified as pre-stress bound if they: 1) show enrichment in control (no stress) conditions; and 2) this enrichment is reduced in stress. Peaks are classified as stress-induced if their enrichment value is higher in stress than in control conditions. Of the top 100 peaks that were identified and classified, the median pre-stress bound peak showed a 60% decrease in binding upon stress, with a standard deviation of 18%. For motif analysis, we searched only those peaks from the top 100 most enriched that are located within 1000 base pairs of a transcription start site (56/100 peaks met this criteria). If multiple peaks in the same promoter could be distinguished, both were included in the analysis.

Promoter scan with Sko1 position weight matrix

Scans of promoter regions for high scoring matches to a position weight matrix were performed using the TestMOTIF software program (Barash *et al.* 2005), using motifs and parameters as described by Tsankov et al. (Tsankov *et al.* 2010). Promoters were defined as 600 base pairs upstream of each open reading frame. (Binding peaks more than 600 base pairs away from a transcription start site were excluded from this analysis.)

Designation of Sko1, Hot1 and Hog1 bound genes

Promoters were designated as Sko1, Hot1 and Hog1 bound (Figure 4, and listed in Table S3) if Hog1 is enriched in that promoter (defined by mock-subtracted Hog1 ChIP signal divided by input for the region 1000 bp upstream of each ORF; promoters are designated as enriched if this value is three standard deviations about the genome-wide median), and Sko1 and Hot1 binding peaks are observed by ChIP-seq. Data from Hot1 ChIP with two different tags was combined to determine Hot1 binding: our genome localization data for HA-tagged Hot1 was determined by ChIP-seq, and previously published ChIP-chip results for TAP-tagged Hot1 (Capaldi *et al.* 2008) was also used as a criteria to identify additional Hot1 bound regions. Both tagged strains show mild to moderate expression defects at some Hot1-regulated genes, suggesting that the tags may affect Hot1 binding or activity (data not shown). For this reason, promoters that display Hot1 binding in either of the two tagged strains are counted as Hot1 bound (Capaldi *et al.* 2008). Two genes (*SCM4* and *HOR2*) with Sko1 and Hot1 binding peaks immediately downstream of the ORF, rather than upstream, are included in the Sko1/Hot1 bound group.

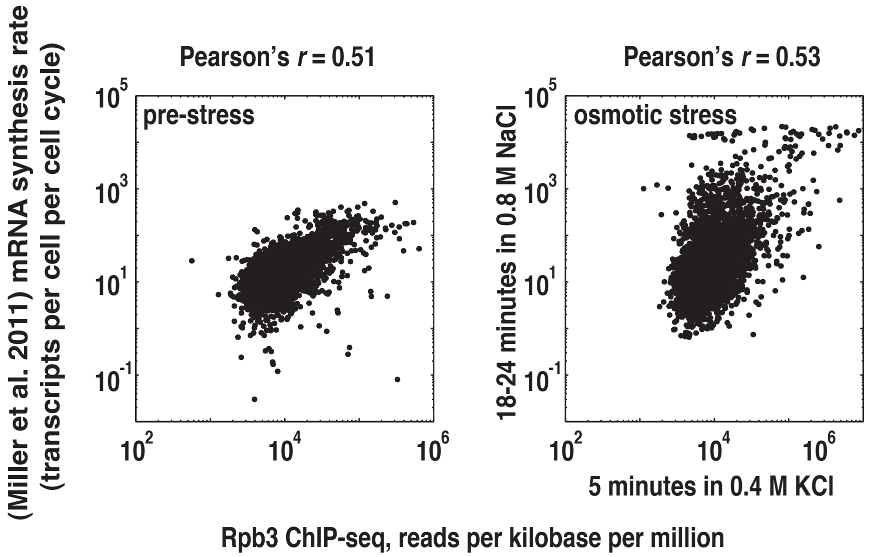

Figure S1 Comparison RNA Pol II occupancy data from this study to data from Miller et al., 2011. (A) Matrix of correlation coefficients between RNA Pol II occupancy measurements obtained in this study to mRNA synthesis rates measured previously (MILLER *et al.* 2011). RNA Pol II occupancy by ChIP-seq in the presence (labeled S) or absence (labeled NS) of osmotic shock, induced by In this study, mRNA synthesis rates were measured in six minute windows by Miller et al. upon induction of osmotic shock by 0.8 M NaCl. (B) Scatter plot comparing RNA Pol II occupancy measurements obtained in this study to mRNA synthesis rates measures by Miller et al., 2011 in the absence of stress (left panel) or upon osmotic shock (right panel; timepoints with the highest correlation are plotted.)

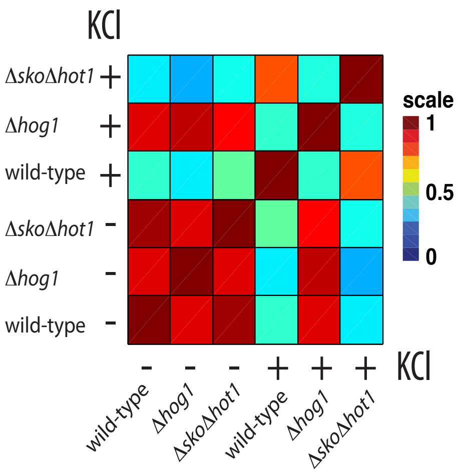

Figure S2 Comparison of RNA Pol II occupancy across ChIP-seq datasets. Matrix shows Pearson correlation coefficients for pair-wise comparisons of genome-wide RNA Pol II occupancy measured by ChIP-seq in wild-type (labeled wt), Δ*hog1*, and Δ*sko1*Δ*hot1* (labeled ΔTF) strains in the presence and absence of osmotic shock by 0.4 M KCl for five minutes. (ORFs with no alignable reads were excluded from the analysis.)

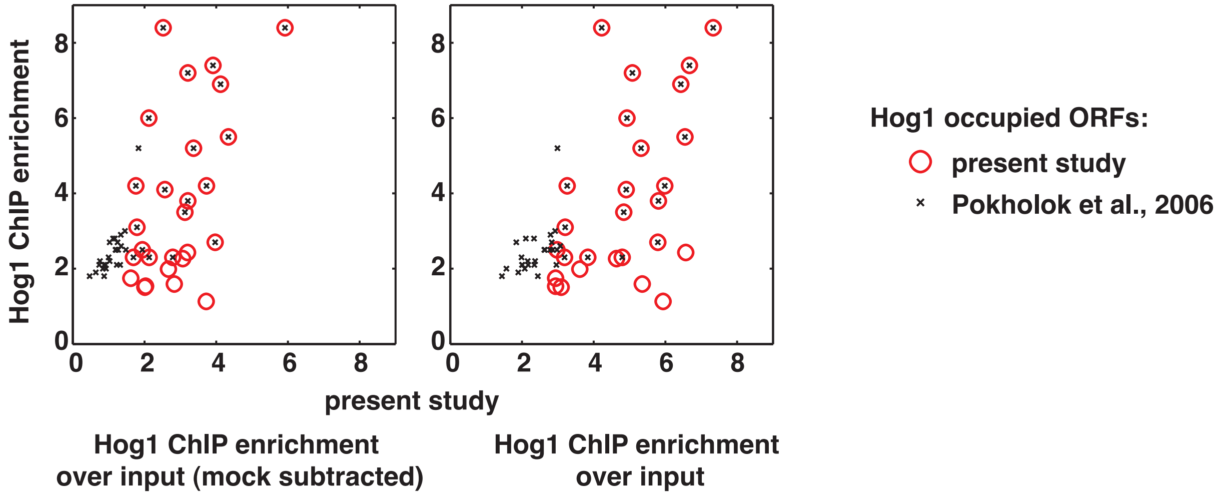

Figure S3 Comparison of Hog1 ORF occupancy data to previous work. Hog1 occupancy measured by ChIP-seq from the present study (osmotic shock was induced with 0.4 M KCl for five minutes; quantities plotted are: mock-subtracted enrichment over input, left panel; or enrichment over input, right panel) plotted against Hog1 occupancy measured by enrichment over input in ChIP-chip (Pokholok *et al.* 2006)during osmotic shock induced by 0.4 M NaCl for five minutes. Each point represents one ORF, and ORFs plotted are those designated as Hog1 occupied by either study.

Table S1 List of strains used in this study

Strain number	genotype
EY0690	*S. cerevisiae* W303
EY2050	*S. cerevisiae* W303, *ade2::ADE2*-P_{MYO2}-rtTA(S2)
EY2281	*S. cerevisiae* W303, *SKO1*::3HA-*SKO1*
EY2290	*S. cerevisiae* W303, *Dhog1::HIS3*
EY2303	*S. cerevisiae* W303, 3HA-*SKO1*, *Dhog1::HIS3*
EY2727	*S. cerevisiae* W303, *HOG1*::*HOG1*-3HA(*HIS3*)
EY2728	*S. cerevisiae* W303, *HOG1*::*HOG1*-3HA(*HIS3*), *Dsko1::LEU2, Dsko1::LEU2, Dhot1::URA3*
EY2735	*S. cerevisiae* W303, *Dhog1::HIS3, ade2::ADE2*-P_{MYO2}-rtTA(S2)
EY2729	*S. cerevisiae* W303,*HOT1*::*HOT1*-3HA(*HIS3*)
EY2730	*S. cerevisiae* W303, *SKO1*::3HA-*sko1-S108A,T113A,S126A*
EB2054	*E. coli* DH5a, contains P_{TETO7}-*LACZ* in YCp50 plasmid, *URA3*
EB2055	*E. coli* DH5a, contains P_{CYC1}-*LACZ* in pCM173 plasmid, *TRP1*
EB2056	*E. coli* DH5a, contains P_{GPD1}-*LACZ* in pCM173 plasmid, *TRP1*
EB2057	*E. coli* DH5a, contains P_{STL1}-*LACZ* in pCM173 plasmid, *TRP1*

Table S2 List of primers used for ChIP-qPCR

target	direction	sequence
ACT1 ORF	forward	GCCTTCTACGTTTCCATCCA
ACT1 ORF	reverse	AGCGGTTTGCATTTCTTGTT
ADH1 ORF	forward	CAACAATACGCTACCGCTGA
ADH1 ORF	reverse	ACGGTGATACCAGCACACAA
GPD1 ORF	forward	GTTGTGGTTTCGTCGAAGGT
GPD1 ORF	reverse	TTAAACCTTGAGCGGATTGG
ILV5 ORF	forward	TGAACAAGTTCTTGCCTGGA
ILV5 ORF	reverse	GAGAGACAACGGTTTGAACG
LACZ ORF	forward	GCTGGTCACTTCGATGGTTT
LACZ ORF	reverse	TTGGCGGTTTCGCTAAATAC
MYO4 ORF	forward	CATTCTCATGGCGCTTTGTA
MYO4 ORF	reverse	ATGGCGGCAGTAATTATCCA
PDC1 ORF	forward	GGCAATACCGTTCAAAGCAG
PDC1 ORF	reverse	CTTACGCCGCTGATGGTTAC
POL1 ORF	forward	CGTTGGATTCACCGATACCT
POL1 ORF	reverse	TGCCAGTGCAGCTAAACCTA
RHR2 ORF	forward	AAGTTCGCTCCAGACTTTGC
RHR2 ORF	reverse	CAACTTGACAGCACCTGGAA
RTC3 ORF	forward	CAAGCCAATGTTCTTTAAACTCAA
RTC3 ORF	reverse	TTTTGTGTATGCGATGGTTTTC
RTC3 promoter	forward	AAGATTTCCCGTTGCGCTAT
RTC3 promoter	reverse	GGAGAAGAGACACGGAGTAGGA
STL1 ORF	forward	CAGTCACTGGGGACTTACGG
STL1 ORF	reverse	AGACTTGCCATCAACCCTTG
STL1 promoter	forward	CCGTTGTCCCACTATTCCAC
STL1 promoter	reverse	AGGACAAAGTCGGACCCTTC
sub-telomeric region	forward	CCCAGGTACGAAACGCTAAG
sub-telomeric region	reverse	ATAAGGTTGTCGACGGTTGC
TDH3 ORF	forward	AAGAACCCCATGGCAAGTTA
TDH3 ORF	reverse	CTGGTGAAGTTTCCCACGAT

Table S3   List of genes with Sko1, Hot1 and Hog1 present in regulatory regions.

gene	ORF
ALD6	YPL061W
CTT1	YGR088C
FAA1	YOR317W
FMP43	YGR243W
FMP45	YDL222C
FMP48	YGR052W
GPD1	YDL022W
GRE2	YOL151W
HOR2	YER062C
HOR7	YMR251W-A
HSP12	YFL014W
HXT1	YHR094C
NCE102	YPR149W
PIL1	YGR086C
PRM10	YJL108C
RHR2	YIL053W
RTC3	YHR087W
SCM4	YGR049W
STL1	YDR536W
YJL107C	YJL107C

List of genes with Sko1, Hot1 and Hog1 present in regulatory regions. Promoters were designated as Sko1, Hot1 and Hog1 bound if Hog1 is enriched in the promoter and Sko1 and Hot1 binding peaks in regulatory regions are observed by ChIP-seq.

Files S3 and S4

Supporting Data

Available for download as Excel files at http://www.g3journal.org/lookup/suppl/doi:10.1534/g3.112.003251/-/DC1.

File S3 RNA Pol II and Hog1 occupancy data from ChIP-seq. Occupancy data for RNA Pol II, measured by Rpb3 ChIP-seq, in wild-type, *Δhog1*, and *Δsko1Δhot1* strains in the presence and absence of stress, and occupancy data for Hog1, measured by ChIP-seq, in wild-type and *Δsko1Δhot1* strains in the presence and absence of stress. Values listed in table are the number of sequencing reads that align within each ORF, expressed in reads per kilobase per million (RPKM). Where two ORFs overlap, we exclude the overlapping portion from analysis and adjust the ORF length accordingly.

File S4 Motifs for stress-induced binding of Sko1 and Hot1. File contains a position specific scoring matrix (PSSM) for Sko1 and Hot1 stress-induced binding, and a list of high-scoring ($P<0.01$) matches to each motif.

LITERATURE CITED

BARASH, Y., G. ELIDAN, T. KAPLAN and N. FRIEDMAN, 2005 CIS: compound importance sampling method for protein-DNA binding site p-value estimation. Bioinformatics 21: 596-600.

CAPALDI, A. P., T. KAPLAN, Y. LIU, N. HABIB, A. REGEV *et al.*, 2008 Structure and function of a transcriptional network activated by the MAPK Hog1. Nature genetics 40: 1300-1306.

FORDYCE, P. M., D. GERBER, D. TRAN, J. ZHENG, H. LI *et al.*, 2010 De novo identification and biophysical characterization of transcription-factor binding sites with microfluidic affinity analysis. Nature biotechnology 28: 970-975.

HARBISON, C. T., D. B. GORDON, T. I. LEE, N. J. RINALDI, K. D. MACISAAC *et al.*, 2004 Transcriptional regulatory code of a eukaryotic genome. Nature 431: 99-104.

JOHNSON, D. S., A. MORTAZAVI, R. M. MYERS and B. WOLD, 2007 Genome-wide mapping of in vivo protein-DNA interactions. Science 316: 1497-1502.

MILLER, C., B. SCHWALB, K. MAIER, D. SCHULZ, S. DUMCKE *et al.*, 2011 Dynamic transcriptome analysis measures rates of mRNA synthesis and decay in yeast. Molecular systems biology 7: 458.

NEHLIN, J. O., M. CARLBERG and H. RONNE, 1992 Yeast SKO1 gene encodes a bZIP protein that binds to the CRE motif and acts as a repressor of transcription. Nucleic acids research 20: 5271-5278.

POKHOLOK, D. K., J. ZEITLINGER, N. M. HANNETT, D. B. REYNOLDS and R. A. YOUNG, 2006 Activated signal transduction kinases frequently occupy target genes. Science 313: 533-536.

PROFT, M., and K. STRUHL, 2002 Hog1 kinase converts the Sko1-Cyc8-Tup1 repressor complex into an activator that recruits SAGA and SWI/SNF in response to osmotic stress. Molecular cell 9: 1307-1317.

ROBERTSON, G., M. HIRST, M. BAINBRIDGE, M. BILENKY, Y. ZHAO *et al.*, 2007 Genome-wide profiles of STAT1 DNA association using chromatin immunoprecipitation and massively parallel sequencing. Nature methods 4: 651-657.

TSANKOV, A. M., D. A. THOMPSON, A. SOCHA, A. REGEV and O. J. RANDO, 2010 The role of nucleosome positioning in the evolution of gene regulation. PLoS biology 8: e1000414.
