# Supplementary material for: Hog1 Controls Global Reallocation of RNA Pol II upon Osmotic Shock in Saccharomyces cerevisiae
Source: G3 (Bethesda). 2012 Sep 1;2(9):1129–36. doi: 10.1534/g3.112.003251 (PMC3429927; doi:10.1534/g3.112.003251)
Supplement: Supporting Information [file supp_2.9.1129_FileS1.pdf]

File S1

Supporting Results and Discussion

Stress-induced Sko1 binding predicts Hog1 ORF occupancy

During osmotic shock, the Hog1-regulated transcription factor Sko1 is found in the promoters of 27 of the 28 Hog1 occupied ORFs. Sko1 recognizes a well-defined consensus motif *in vivo* in the absence of stress (CAPALDI *et al.* 2008; HARBISON *et al.* 2004), and binds to this same motif *in vitro* (FORDYCE *et al.* 2010; NEHLIN *et al.* 1992). However, the Sko1 consensus motif is absent (evaluated at $P<0.05$) from 20 of the 27 Sko1-bound promoters of Hog1-occupied ORFs. Sko1 was once thought to be constitutively bound to its target promoters, and depending on cellular conditions, recruit either repressive or activating chromatin remodeling factors (PROFT and STRUHL 2002). However, a genome-wide study of Sko1 binding behavior demonstrates that Sko1 target promoters do not display uniform binding behavior. Instead, genes with Sko1 bound in the promoter fall into two basic classes (CAPALDI *et al.* 2008). At one set of genes, Sko1 is bound in normal growth conditions and partially released from the promoter in response to osmotic stress, while for another set of genes, Sko1 binding enrichment increases in response to stress. By using ChIP-seq to generate a high-resolution map of Sko1 binding, we are able to identify individual binding sites that fall into these two classes. We find that stress-induced Sko1 binding correlates with the presence of Hog1 in ORFs, while pre-stress Sko1 binding does not. The presence of a stress-induced Sko1 binding peak in a promoter is predictive of Hog1 presence in promoters and ORFs; genes with stress-induced Sko1 promoter binding peaks show a median Hog1 enrichment (defined by mock-subtracted Hog1 ChIP-seq signal, divided by input) of 5.0 in promoters and 2.8 in ORFs during osmotic shock. In contrast, pre-stress binding of Sko1 in promoters is not predictive of Hog1 presence in promoters (median enrichment of 1.26) or ORFs (median enrichment 0.6) during stress.
