# Supplementary material for: Hog1 Controls Global Reallocation of RNA Pol II upon Osmotic Shock in Saccharomyces cerevisiae
Source: G3 (Bethesda). 2012 Sep 1;2(9):1129–36. doi: 10.1534/g3.112.003251 (PMC3429927; doi:10.1534/g3.112.003251)
Supplement: Supporting Information [file supp_2.9.1129_FileS2.pdf]

File S2

Supporting Materials and Methods

Chromatin Immunoprecipitation

ChIP-seq and ChIP-qPCR experiments were conducted as described previously (CAPALDI *et al.* 2008; JOHNSON *et al.* 2007; ROBERTSON *et al.* 2007). Cells were diluted from an overnight culture to an OD_{600} of 0.1. Cells were grown at 30° C in YEPD, with shaking, to OD_{600} of 0.6. Cultures were split for stress treatment and mock treatment. For stress treatment, YEPD supplemented with KCl was added to ~120 OD units of culture, bringing the final concentration of KCl to 0.4 M; for mock treated cells, the same volume of YEPD was added to cultures. After five minutes in stress, samples were crosslinked with 1% formaldehyde at room temperature for 15 minutes. Crosslinking was quenched with 125 mM glycine for five minutes, and then samples were harvested by centrifugation, washed twice in cold PBS (137 mM NaCl, 2.7 mM KCl, 10 mM sodium phosphate dibasic, 2 mM potassium phosphate monobasic, pH 7.4) and then snap-frozen in liquid nitrogen. Samples were resuspended in 1 mL lysis buffer (50 mM HEPES pH 7.5, 140 mM NaCl, 1 mM EDTA, 1% Triton-X 100, 0.1% Na Deoxycholate) in the presence of protease inhibitors (Roche; Complete) and mechanically lysed by bead beading. Lysates were sonicated (9 cycles of 15 seconds each, power 2 on Misonix 3000) to solubilize chromatin and then clarified by centrifugation. Ten percent of the clarified lysate was reserved to serve as an input control. Clarified lysates (~10 mg for ChIP-seq, ~ 2.5 mg for ChIP qPCR) were incubated with 12CA5 anti-HA antibody, for 2 hours at 4° C before addition of Protein G Dynabeads (Invitrogen). After incubation for 4 hours up to overnight, beads were washed twice with lysis buffer, once with high salt buffer (50 mM HEPES pH 7.5, 500 mM NaCl, 1 mM EDTA, 1% Triton-X 100, 0.1% Na Deoxycholate), once with lysis buffer and once with TE (10 mM Tris, 1 mM EDTA, pH 8). All washes were performed at room temperature for five minutes each, on an end-over-end mixer. Samples were eluted from beads in TE plus 0.67% SDS at 65° C for 30 minutes. Supernatants were removed from beads and incubated overnight at 65° C to break crosslinks. DNA was isolated by digestion of RNA with RNAse A for 2 hours at 37° C, protein digestion with Proteinase K for 2 hours at 55° C, and purified by phenol chloroform extraction and precipitation with ethanol and NaCl. DNA pellets were stored in TE.

For ChIP-seq, ~ 10 ng IP material was used to generate each library, following the Illumina protocol for their paired end DNA sample prep kit (v1). After addition of adaptors, DNA in a size range of 175-300 base pairs was isolated by gel electrophoresis for amplification. Size ranges of prepared libraries were measured on an Agilent Biolanalyzer (average size 225 base pairs) before sequencing on an Illumina Genome Analyzer II (performed by Christian Daly at the FAS Center for Systems Biology Core Facility). Thirty-six base reads were obtained and aligned to the *Saccharomyces cerevisiae* genome using ELAND (Jianwen Zhang performed the alignments). For Hog1 and transcription factor ChIP-seq samples, five million to ten million

reads were obtained. Uniquely alignable sequence tags were mapped to the genome and extended by the average length of the library (minus the adaptor length) in MATLAB. For ChIP-qPCR, samples were analyzed on an MX3000p qPCR machine (Stratagene) using primers that amplify a ~ 100 base pair region surrounding the center of observed binding peaks, or an ~300 base pair region in the coding sequence of the ORF. The sequences of these qPCR primers are listed in Supplemental Table S2.

Identification of stress-induced and pre-stress bound Sko1 binding peaks

Sko1 binding peaks were classified as pre-stress bound if they: 1) show enrichment in control (no stress) conditions; and 2) this enrichment is reduced in stress. Peaks are classified as stress-induced if their enrichment value is higher in stress than in control conditions. Of the top 100 peaks that were identified and classified, the median pre-stress bound peak showed a 60% decrease in binding upon stress, with a standard deviation of 18%. For motif analysis, we searched only those peaks from the top 100 most enriched that are located within 1000 base pairs of a transcription start site (56/100 peaks met this criteria). If multiple peaks in the same promoter could be distinguished, both were included in the analysis.

Promoter scan with Sko1 position weight matrix

Scans of promoter regions for high scoring matches to a position weight matrix were performed using the TestMOTIF software program (BARASH *et al.* 2005), using motifs and parameters as described by Tsankov et al. (TSANKOV *et al.* 2010). Promoters were defined as 600 base pairs upstream of each open reading frame. (Binding peaks more than 600 base pairs away from a transcription start site were excluded from this analysis.)

Designation of Sko1, Hot1 and Hog1 bound genes

Promoters were designated as Sko1, Hot1 and Hog1 bound (Figure 4, and listed in Table S3) if Hog1 is enriched in that promoter (defined by mock-subtracted Hog1 ChIP signal divided by input for the region 1000 bp upstream of each ORF; promoters are designated as enriched if this value is three standard deviations about the genome-wide median), and Sko1 and Hot1 binding peaks are observed by ChIP-seq. Data from Hot1 ChIP with two different tags was combined to determine Hot1 binding: our genome localization data for HA-tagged Hot1 was determined by ChIP-seq, and previously published ChIP-chip results for TAP-tagged Hot1 (CAPALDI *et al.* 2008) was also used as a criteria to identify additional Hot1 bound regions. Both tagged strains show mild to moderate expression defects at some Hot1-regulated genes, suggesting that the tags may affect Hot1 binding or activity (data not shown). For this reason, promoters that display Hot1 binding in either of the two tagged strains are counted as Hot1 bound (CAPALDI *et al.* 2008). Two genes (*SCM4* and *HOR2*) with Sko1 and Hot1 binding peaks immediately downstream of the ORF, rather than upstream, are included in the Sko1/Hot1 bound group.
