# Supplementary material for: Hog1 Controls Global Reallocation of RNA Pol II upon Osmotic Shock in Saccharomyces cerevisiae
Source: G3 (Bethesda). 2012 Sep 1;2(9):1129–36. doi: 10.1534/g3.112.003251 (PMC3429927; doi:10.1534/g3.112.003251)
Supplement: Supporting Information [file supp_2.9.1129_FigureS1.pdf]

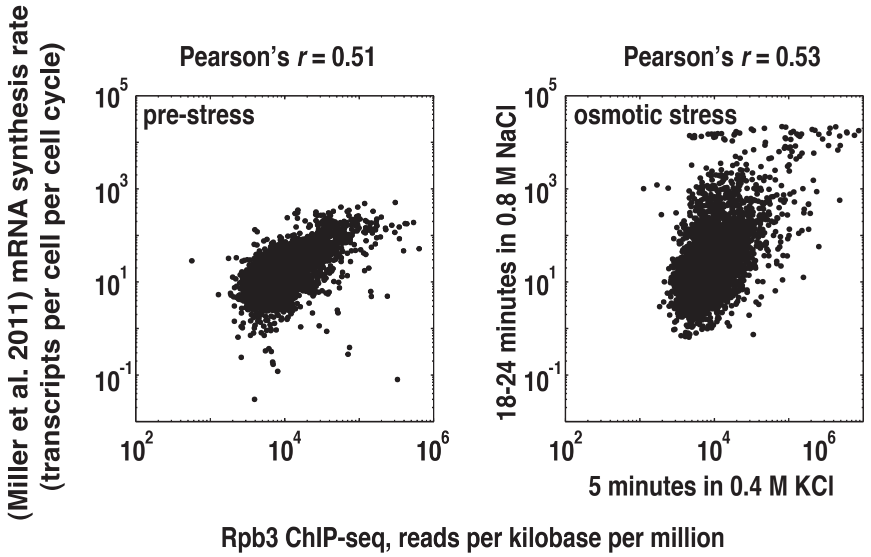

Figure S1 Comparison RNA Pol II occupancy data from this study to data from Miller et al., 2011. (A) Matrix of correlation coefficients between RNA Pol II occupancy measurements obtained in this study to mRNA synthesis rates measured previously (MILLER *et al.* 2011). RNA Pol II occupancy by ChIP-seq in the presence (labeled S) or absence (labeled NS) of osmotic shock, induced by In this study, mRNA synthesis rates were measured in six minute windows by Miller et al. upon induction of osmotic shock by 0.8 M NaCl. (B) Scatter plot comparing RNA Pol II occupancy measurements obtained in this study to mRNA synthesis rates measures by Miller et al., 2011 in the absence of stress (left panel) or upon osmotic shock (right panel; timepoints with the highest correlation are plotted.)
