# Supplementary material for: Hog1 Controls Global Reallocation of RNA Pol II upon Osmotic Shock in Saccharomyces cerevisiae
Source: G3 (Bethesda). 2012 Sep 1;2(9):1129–36. doi: 10.1534/g3.112.003251 (PMC3429927; doi:10.1534/g3.112.003251)
Supplement: Supporting Information [file supp_2.9.1129_FigureS2.pdf]

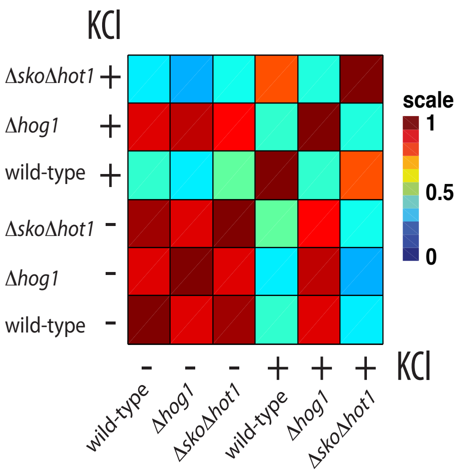

Figure S2 Comparison of RNA Pol II occupancy across ChIP-seq datasets. Matrix shows Pearson correlation coefficients for pair-wise comparisons of genome-wide RNA Pol II occupancy measured by ChIP-seq in wild-type (labeled wt), Δ*hog1*, and Δ*sko1*Δ*hot1* (labeled ΔTF) strains in the presence and absence of osmotic shock by 0.4 M KCl for five minutes. (ORFs with no alignable reads were excluded from the analysis.)
