# Supplementary material for: Hog1 Controls Global Reallocation of RNA Pol II upon Osmotic Shock in Saccharomyces cerevisiae
Source: G3 (Bethesda). 2012 Sep 1;2(9):1129–36. doi: 10.1534/g3.112.003251 (PMC3429927; doi:10.1534/g3.112.003251)
Supplement: Supporting Information [file supp_2.9.1129_FigureS3.pdf]

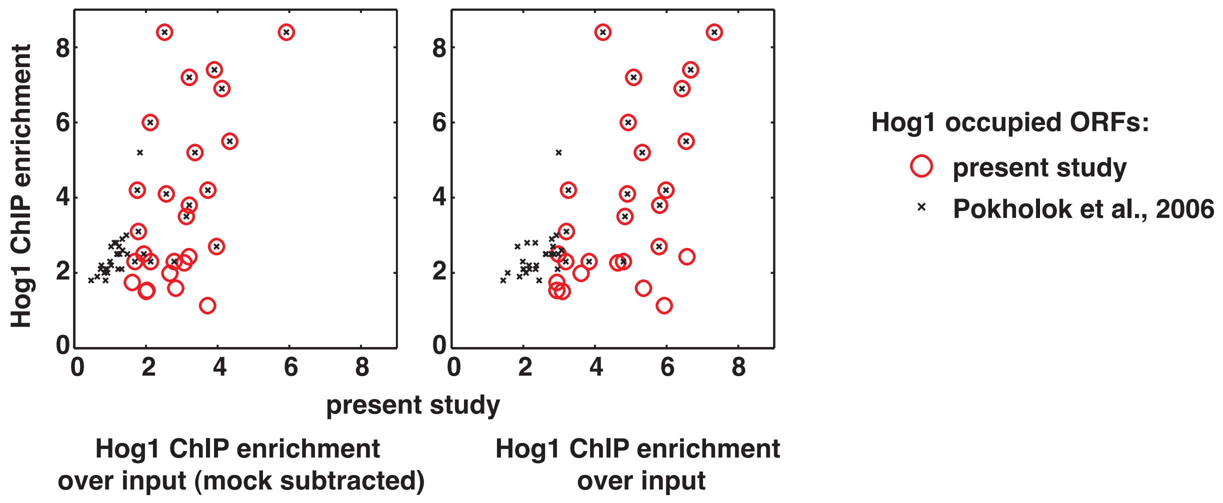

Figure S3 Comparison of Hog1 ORF occupancy data to previous work. Hog1 occupancy measured by ChIP-seq from the present study (osmotic shock was induced with 0.4 M KCl for five minutes; quantities plotted are: mock-subtracted enrichment over input, left panel; or enrichment over input, right panel) plotted against Hog1 occupancy measured by enrichment over input in ChIP-chip (Pokholok *et al.* 2006)during osmotic shock induced by 0.4 M NaCl for five minutes. Each point represents one ORF, and ORFs plotted are those designated as Hog1 occupied by either study.
