# Supplementary material for: Hog1 Controls Global Reallocation of RNA Pol II upon Osmotic Shock in Saccharomyces cerevisiae
Source: G3 (Bethesda). 2012 Sep 1;2(9):1129–36. doi: 10.1534/g3.112.003251 (PMC3429927; doi:10.1534/g3.112.003251)
Supplement: Supporting Information [file supp_2.9.1129_TableS1.pdf]

Table S1 List of strains used in this study

Strain number	genotype
EY0690	*S. cerevisiae* W303
EY2050	*S. cerevisiae* W303, *ade2::ADE2*-P_{MYO2}-rtTA(S2)
EY2281	*S. cerevisiae* W303, *SKO1::*3HA-*SKO1*
EY2290	*S. cerevisiae* W303, *Dhog1::HIS3*
EY2303	*S. cerevisiae* W303, 3HA-*SKO1*, *Dhog1::HIS3*
EY2727	*S. cerevisiae* W303, *HOG1*::*HOG1*-3HA(*HIS3*)
EY2728	*S. cerevisiae* W303, *HOG1*::*HOG1*-3HA(*HIS3*), *Dsko1::LEU2, Dsko1::LEU2, Dhot1::URA3*
EY2735	*S. cerevisiae* W303, *Dhog1::HIS3, ade2::ADE2*-P_{MYO2}-rtTA(S2)
EY2729	*S. cerevisiae* W303,*HOT1*::*HOT1*-3HA(*HIS3*)
EY2730	*S. cerevisiae* W303, *SKO1::*3HA-*sko1-S108A,T113A,S126A*
EB2054	*E. coli* DH5a, contains P_{TETO7}-*LACZ* in YCp50 plasmid, *URA3*
EB2055	*E. coli* DH5a, contains P_{CYC1}-*LACZ* in pCM173 plasmid, *TRP1*
EB2056	*E. coli* DH5a, contains P_{GPD1}-*LACZ* in pCM173 plasmid, *TRP1*
EB2057	*E. coli* DH5a, contains P_{STL1}-*LACZ* in pCM173 plasmid, *TRP1*
