# Supplementary material for: Hog1 Controls Global Reallocation of RNA Pol II upon Osmotic Shock in Saccharomyces cerevisiae
Source: G3 (Bethesda). 2012 Sep 1;2(9):1129–36. doi: 10.1534/g3.112.003251 (PMC3429927; doi:10.1534/g3.112.003251)
Supplement: Supporting Information [file supp_2.9.1129_TableS2.pdf]

Table S2 List of primers used for ChIP-qPCR

target	direction	sequence
ACT1 ORF	forward	GCCTTCTACGTTTCCATCCA
ACT1 ORF	reverse	AGCGGTTTGCATTTCTTGTT
ADH1 ORF	forward	CAACAATACGCTACCGCTGA
ADH1 ORF	reverse	ACGGTGATACCAGCACACAA
GPD1 ORF	forward	GTTGTGGTTTCGTCGAAGGT
GPD1 ORF	reverse	TTAAACCTTGAGCGGATTGG
ILV5 ORF	forward	TGAACAAGTTCTTGCCTGGA
ILV5 ORF	reverse	GAGAGACAACGGTTTGAACG
LACZ ORF	forward	GCTGGTCACTTCGATGGTTT
LACZ ORF	reverse	TTGGCGGTTTCGCTAAATAC
MYO4 ORF	forward	CATTCTCATGGCGCTTTGTA
MYO4 ORF	reverse	ATGGCGGCAGTAATTATCCA
PDC1 ORF	forward	GGCAATACCGTTCAAAGCAG
PDC1 ORF	reverse	CTTACGCCGCTGATGGTTAC
POL1 ORF	forward	CGTTGGATTCACCGATACCT
POL1 ORF	reverse	TGCCAGTGCAGCTAAACCTA
RHR2 ORF	forward	AAGTTCGCTCCAGACTTTGC
RHR2 ORF	reverse	CAACTTGACAGCACCTGGAA
RTC3 ORF	forward	CAAGCCAATGTTCTTTAAACTCAA
RTC3 ORF	reverse	TTTTGTGTATGCGATGGTTTTC
RTC3 promoter	forward	AAGATTTCCCGTTGCGCTAT
RTC3 promoter	reverse	GGAGAAGAGACACGGAGTAGGA
STL1 ORF	forward	CAGTCACTGGGGACTTACGG
STL1 ORF	reverse	AGACTTGCCATCAACCCTTG
STL1 promoter	forward	CCGTTGTCCCACTATTCCAC
STL1 promoter	reverse	AGGACAAAGTCGGACCCTTC
sub-telomeric region	forward	CCCAGGTACGAAACGCTAAG
sub-telomeric region	reverse	ATAAGGTTGTCGACGGTTGC
TDH3 ORF	forward	AAGAACCCCATGGCAAGTTA
TDH3 ORF	reverse	CTGGTGAAGTTTCCCACGAT
