# Supplementary material for: Hog1 Controls Global Reallocation of RNA Pol II upon Osmotic Shock in Saccharomyces cerevisiae
Source: G3 (Bethesda). 2012 Sep 1;2(9):1129–36. doi: 10.1534/g3.112.003251 (PMC3429927; doi:10.1534/g3.112.003251)
Supplement: Supporting Information [file supp_2.9.1129_TableS3.pdf]

**Table S3 List of genes with Sko1, Hot1 and Hog1 present in regulatory regions.**

| gene | ORF |
|---|---|
| ALD6 | YPL061W |
| CTT1 | YGR088C |
| FAA1 | YOR317W |
| FMP43 | YGR243W |
| FMP45 | YDL222C |
| FMP48 | YGR052W |
| GPD1 | YDL022W |
| GRE2 | YOL151W |
| HOR2 | YER062C |
| HOR7 | YMR251W-A |
| HSP12 | YFL014W |
| HXT1 | YHR094C |
| NCE102 | YPR149W |
| PIL1 | YGR086C |
| PRM10 | YJL108C |
| RHR2 | YIL053W |
| RTC3 | YHR087W |
| SCM4 | YGR049W |
| STL1 | YDR536W |
| YJL107C | YJL107C |

List of genes with Sko1, Hot1 and Hog1 present in regulatory regions. Promoters were designated as Sko1, Hot1 and Hog1 bound if Hog1 is enriched in the promoter and Sko1 and Hot1 binding peaks in regulatory regions are observed by ChIP-seq.
